# Are competing risks models appropriate to describe implant failure?

**DOI:** 10.1080/17453674.2018.1452470

**Published:** 2018-05-04

**Authors:** Jonas Ranstam

**Affiliations:** Statistics Editor

In this issue of *Acta Orthopaedica*, Sayers et al. raise the issue of whether competing risks models are appropriate to describe implant failure. The question is relevant because the number of publications presenting competing risks models is increasing rapidly. A PubMed search with the criteria “arthroplasty” and “competing risk” reveals that while no such paper was published before 2000, all but 2 of 46 published papers were published in 2010 or later, and of these not less than 17 were published during 2017 alone. It should thus not come as a surprise if many more papers using competing risks models are published during the coming years.

What is a competing risks model, and why is this suddenly so interesting? First, implant survival has traditionally been analyzed using the Kaplan–Meier method. This allows inclusion also of observations with incomplete follow-up in the analysis; these are known as censored observations. The analysis is, however, problematic in the presence of competing events (such as death) that preclude the studied event (implant failure). This changes the interpretation of the failure-rate estimate. An alternative technique that accounts for the competing event, a competing risks model, is then often recommended.

Sayers et al. have recently noticed several publications in which the differences in results between the Kaplan–Meier method and competing risks models have been misinterpreted. To explain the problem they describe two different measures, net and crude failure: the former is calculated using the Kaplan–Meier method and the latter with a competing risks model.

Both estimates can be useful in arthroplasty studies and both provide, in the absence of confounding and selection bias, unbiased estimates. The main difference between the two measures is that the net failure refers to a hypothetical existence in which competing risks are assumed to be eliminated. In real life, the (crude) failure rate is lower for elderly patients as they are more likely to be excluded from failure because of the competing risk of dying.

Which estimate should be presented in scientifi c publications on implant failure and annual reports from arthroplasty registers? This depends on the application of interest. Net failure is the relevant measure when comparing the failure rates of different implants. It would clearly not be reasonable to include effects of patient survival in this comparison. Crude failure, on the other hand, is the relevant measure if patient survival is part of the problem, as for example when studying health economics and planning resources.

An important aspect of the paper by Sayers et al. is that it draws attention to the general importance of recognizing why a study is performed and to the limitations in using the results for other purposes. Knowledge of a study’s aim, design, and analysis is usually necessary for a correct interpretation of its findings.

## References

[C1] Sayers A, Evans J T, Whitehouse M R, Blom A W. Are competing risk models appropriate to describe implant failure? Acta Orthop 2018; 89 [Ahead of print] DOI: 10.1080/17453674.2018.1444876 29521152 PMC6055780

